# Incorporating one health into medical education

**DOI:** 10.1186/s12909-017-0883-6

**Published:** 2017-02-23

**Authors:** Peter M. Rabinowitz, Barbara J. Natterson-Horowitz, Laura H. Kahn, Richard Kock, Marguerite Pappaioanou

**Affiliations:** 1 Departments of Environmental and Occupational Health Sciences, Global Health, Family Medicine, University of Washington Center for One Health Research, 1959 NE Pacific Street HSB F551, Box 357234, Seattle, WA 98195 USA; 2 Division of Cardiology, David Geffen School of Medicine, University of California, Los Angeles, USA; 3 Program on Science and Global Security, Woodrow Wilson School of Public and International Affairs, Princeton University, Princeton, USA; 4 Department of Pathology and Pathogen Biology, Royal Veterinary College, University of London, London, UK; 5 Centers for Disease Control and Prevention Liaison to the Food and Drug Administration for Food Safety, Washington, DC USA

**Keywords:** One Health, Medical education, Zoonoses, Human-animal bond, Interdisciplinary education, Environmental health

## Abstract

One Health is an emerging concept that stresses the linkages between human, animal, and environmental health, as well as the need for interdisciplinary communication and collaboration to address health issues including emerging zoonotic diseases, climate change impacts, and the human-animal bond. It promotes complex problem solving using a systems framework that considers interactions between humans, animals, and their shared environment. While many medical educators may not yet be familiar with the concept, the One Health approach has been endorsed by a number of major medical and public health organizations and is beginning to be implemented in a number of medical schools. In the research setting, One Health opens up new avenues to understand, detect, and prevent emerging infectious diseases, and also to conduct translational studies across species. In the clinical setting, One Health provides practical ways to incorporate environmental and animal contact considerations into patient care. This paper reviews clinical and research aspects of the One Health approach through an illustrative case updating the biopsychosocial model and proposes a basic set of One Health competencies for training and education of human health care providers.

## Background

The conceptual model under which physicians train affects the way they approach patient care. Changing patterns in diseases on a global scale suggest a need for new conceptual models for medical education. Rapid global population growth and mobility, agricultural intensification, and the effects of accelerating climate change are impacting biodiversity and ecosystems challenging planetary boundaries for sustainability and creating new environmental health threats at the community and individual level [1]. Emerging infectious diseases in recent decades, driven in large part by such environmental developments, are mostly zoonotic (transmitted between animals and humans) in origin. Zoonotic disease outbreaks and pandemics including Severe Acute Respiratory Syndrome (SARS), avian influenza, pandemic 2009 H1N1 influenza, West Nile virus, Middle East Respiratory Syndrome (MERS), and Ebola, are occurring with increasing frequency and threaten global health security and economic stability [2, 3]. There is increasing evidence for the changing relationship with our environment leading to many chronic and emerging challenges such as malnutrition associated with food systems leading to both under- and over nutrition, and diseases relating to declining quality of the environment in particular the air, water, soils and access to space and nature. Antibiotic resistant bacteria are emerging in both humans and animals related to widespread use of antibiotics across species including in animal agriculture [4]. At the same time, studies of physicians reveal limited awareness of the environmental health aspects of medical problems in the patient care setting [5], as well as low levels of awareness about prevention or treatment of zoonotic diseases [6]. Therefore, there have been calls for training health professionals in “systems thinking” to better prepare them to face these emerging disease issues [7].

**Fig. 1:**
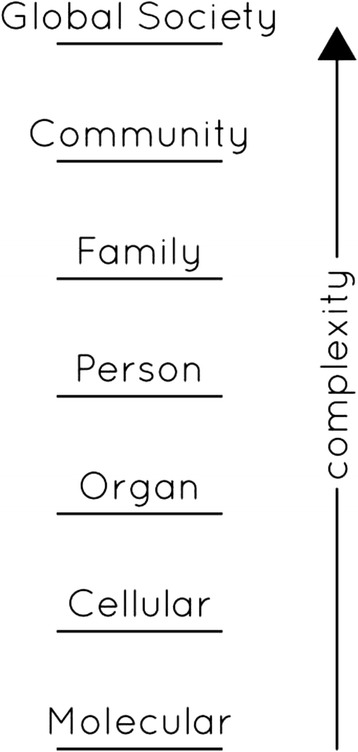
The systems hierarchy of the biopsychosocial model (BPS)

A precedent for teaching systems approaches in medical education can be found in the development of Engel’s biopsychosocial model. In 1980, Engel presented a case of a patient with chest pain to demonstrate how a reductionist “biomedical” model (that breaks down biological processes into discrete pathways and considers each separately [8] could miss important elements of the social and psychological aspects of care. The “biopsychosocial” model for medical care that he proposed as an alternative approach to a clinical situation was a systems approach that considers the patient as part of a larger system with hierarchical levels of increasing complexity, from the molecular to the cellular to the organ, individual, community, and society (Fig. 1) [9, 10]. The concepts of patient-centered care and consideration of social determinants of health can be seen as the most recent efforts to incorporate biopsychosocial approaches into the patient-provider encounter [11, 12]. It now seems timely to adapt and update the biopsychosocial model into a “One Health” framework that applies the same type of systems thinking to disease challenges in a rapidly changing global environment.

## Main text

### The one health concept- research and clinical aspects

**Fig. 2:**
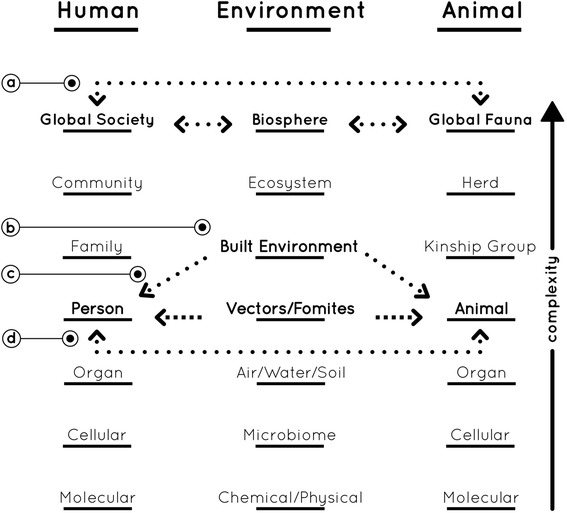
One Health: interconnected human, environment, and animal health systems: arrows refer to examples in the illustrative case. **a** *Arrow* indicates “Planetary level” interactions of climate (such as heat waves) on global populations of humans and animals. **b** *Arrow* indicates that both the people and animals in the neighborhood are affected by the shared built environment, including degree of heat stress related to amount of neighborhood greenspace and pavement. **c** *Arrow* indicates shared exposure of Mr. Glover and his dog to household built environment aspects including building design and ventilation as well as the presence of ticks and other vectors in the immediate vicinity. **d** *Arrow* indicates direct clinical relationships between Mr. Glover and his dog, including the human animal bond and comparisons between his disease manifestations and that of the dog

The One Health concept recognizes health connections between humans, animals, and their shared environments [13]. It promotes professional cooperation between physicians, veterinarians, and others to address complex problems affecting multiple species and pathogens in changing environments. Like Engel’s Biopsychosocial model, the One Health model is a systems framework, but one that considers animal and environmental as well as human health systems (Fig. 2). The concept has been endorsed by a number of national and state medical organizations and international agencies including the American Medical Association, the Centers for Disease Control, and the World Health Organization [14]. There have been calls for academic medical institutions to adopt One Health transdisciplinary approaches to research and education [15, 16]. Student One Health interest groups have been formed in a number of medical schools, and some academic health centers have reorganized existing programs and initiated new ones around One Health principles [17].

One Health stresses the need to address zoonoses and other emerging disease threats by considering factors affecting the balance, health, and stability of supporting ecosystems, including climate change, agricultural intensification, food systems and wildlife habitat destruction [18, 19]. Current One Health research efforts include a global surveillance initiative to identify new pathogens with pandemic potential in wildlife animal reservoirs and the ecological factors driving zoonotic emergence [20]. Such work could enable the development of vaccines, diagnostic methods, and other measures to prevent future pandemics.

While useful for addressing the problems of zoonotic disease emergence, the One Health concept extends far beyond infectious disease to include overlaps between human and animal health such as the human-animal bond. More than 50% of US households have pets that are increasingly considered part of the family, and growing evidence suggests positive mental and physical health benefits of human-animal contact [21]. Active areas of research related to the human animal bond include studies of the therapeutic effect of contact with animals in the clinical setting [22], and determining the benefits and risks of companion animals in the home [23].

One Health also considers the potential for physicians and veterinarians to learn from each other through a comparative, evolutionary approach to disease and health [24]. For example, psychiatrists may derive insights into the causes of anxiety, eating disorders, and self-injury by learning about the spontaneous occurrence of these disorders in non-human animals. Similarly, medical oncologists may derive insights into treatments by learning about the types of cancers that occur in mammals and other species, and how veterinarians are treating these cancers in multiple species [25]. Increasingly, clinical trials in companion animals such as dogs are becoming part of the process of developing and testing drugs that could benefit both humans as well as other species [26].

### Clinical case example

The following case, adapted from Engel [9] illustrates how a One Health approach can address clinically relevant interactions between a patient, animals in contact with that patient, and their shared environment.

Mr. Glover, a 55-year-old real estate salesman, presents to the emergency room with chest pain that began earlier that day. He has a history of a myocardial infarction six months previously, reports gaining 20 pounds over the past year, and was reluctant to seek care when the pain started.

The emergency medicine resident orders diagnostic studies including an electrocardiogram, chest X-ray, CT scan with a pulmonary embolism protocol, cardiac enzymes, and arterial blood gases. The resident has difficulty obtaining the arterial blood gas and the patient becomes increasingly uncomfortable. After several attempts the patient develops ventricular fibrillation, but is able to be resuscitated.

As the patient is being admitted to the hospital floor, the admitting medical staff elicits a more patient-centered and biopsychosocial history. The patient’s wife is currently out of town, and he had been unable to reach her when his symptoms began that morning. The patient has been working long hours; sleeping poorly, and in general had been in denial about the increasing frequency of his chest pain symptoms in recent days. He had only agreed to go to the emergency room after his work supervisor had called 911. Now he reports feeling overwhelmed and fearful. Based on this information, his care team contacts his wife and also provides additional counseling to the patient about his medical condition. Despite this, he continues to appear anxious.

### Added value of the one health model

Taking a One Health approach to this patient would include obtaining a more comprehensive history of present illness that considers both relevant environmental exposures as well as contact with pets and other animals. Taking a pet history is feasible in the primary care setting [27] and in the hospital can be incorporated into either the nursing or medical history taking process. If the clinician caring for Mr. Glover in the Emergency Department could take such an animal contact history, it would reveal that he is anxious because his dog, considered an important member of the family, has been left alone at home. The patient is concerned because the weather report has announced a heat illness alert due to an ongoing heat wave, and the air conditioning in the house is not working (this may also be contributing to the patient’s lack of adequate sleep). The dog had been lame for several months and has limited mobility. When Mr. Glover had developed chest pain at work, the chief reason for his reluctance to go to the emergency room, he would now disclose to the clinician, was his concern about leaving his dog alone in the house during the heat advisory expected later in the day.

Mr. Glover’s concern for his pet’s well-being ahead of his own health is typical of many people who in situations of natural disasters [28] and even domestic violence [29] refuse to leave their pets behind. Similarly, concern for a pet has been cited as a reason for patients leaving the hospital against medical advice [30]. At the same time, pets, like the “canary in the coal mine”, can serve as “sentinels” of environmental health hazards to human health. For example, pets, may suffer the effects of heat stress before humans, due in part to due to their inability to sweat effectively [31], and to escape a heat-exposed environment [32]. Similarly, abuse diagnosed in a pet may be a warning sign of the risk of domestic violence to humans in the same household [33]. In this way, clinical information about the health of pets may provide important clues regarding health risks of humans sharing that household environment.

In this case, relevant interactions between human, animal and environment are taking place at the individual level, with the patient and the dog sharing an overheated house due to broken air conditioning. However, it is obvious that human/animal/ecosystem interactions at many other levels are possible (Fig. 2). For example, the amount of green space and pavement in the neighborhood’s built environment may affect the impact of extreme heat events. At a higher, more global or “planetary” level, clinicians trained in a One Health approach could move beyond intuitively obvious causation to consider how the impacts of increasing human and domestic animal populations are contributing to climate change and effects such as extreme heat and other weather events.

The One Health perspective of clinical comparisons between species can be applied to Mr. Glover’s ventricular fibrillation in the setting of a painful emergency procedure. Veterinarians routinely take steps to avoid unnecessary stress on animals and as a profession recognized the condition of “capture myopathy” years before its corollary in humans, Takotsubo’s cardiomyopathy [34], an acute cardiomyopathy induced by stress, was described in the medical literature. Are there lessons to learn from veterinary medicine regarding the care of acutely ill patients that could have prevented this patient’s adverse event?

Other aspects of the health of Mr. Glover’s dog could be relevant to his case. The dog’s lameness and limited mobility could be making Mr. Glover more sedentary by reducing the amount of dog walking activities. A possible cause of the dog’s lameness could be a tick-borne infection such as Lyme [35] or Bartonella [36] that could also pose a risk to humans in the household, with potential clinical manifestations including cardiac disease. Better clinical communication between human and animal health clinicians could improve diagnosis of such zoonotic diseases across species [37].

Applying the One Health model to Mr. Glover’s case could also enable novel interventions. At the individual level, arrangements could be made to have someone go to the house and check on the dog, thereby reducing his anxiety and potentially preventing the entire illness episode. Even after being hospitalized, animal-assisted therapy animals could provide Mr. Glover some comfort and stress reduction; dogs and other animals are increasingly being used to reduce patient stress and isolation in the hospital care setting [38].

Ensuring adequate veterinary care to correctly diagnose and treat the dog could improve the lameness, which would allow Mr. Glover to resume a better exercise schedule. A home visit could deal with environmental issues on a household level, such as fixing the air conditioner to help both human and dog better withstand the next heat wave. At the community level, revised emergency room procedures using ideas borrowed from veterinary medicine could reduce stress for patients. Other community interventions could include increasing greenspace and the walkability of the neighborhood, with both physical and psychological respite benefits to humans and animals [39].

Enhanced communications between human and animal health care providers about these shared health issues could improve awareness of the impact of climate change on the health of their patients. This could lead such professionals to work jointly to mitigate the effects of climate change at the household and local community level, and also to support policy measures addressing the health impacts of climate change at the “planetary level” (Fig. 2).

### Medical education and one health

At present, One Health education efforts in medical schools are in their infancy, and lag behind veterinary schools which have made One Health a central part of their curricula. Recent published initiatives have included inter-professional training between human health and veterinary medical training institutions focusing on topics such as shared access to clean water [40]. Other educational efforts include the development of One Health curricula to educate high school students about infectious diseases [41], and the establishment by the Council for Education in Public Health (CEPH) of the following One Health competency for Masters (MPH) and Doctorates (DrPH) in public health: “Explain an ecological perspective on the connections among human health, animal health and ecosystem health (eg, One Health)” [42].

### Proposed one health competencies for medical education

Wider adoption of the One Health approach to clinical care will require educating and training health care providers in certain novel competencies (Table 1). These proposed competencies involve assessing and managing interactions between patients, animals, and their shared environments. Such interactions include health risks such as zoonoses, allergies, and animal injuries, but also health benefits including the human animal bond and the ability of animals to serve as sentinels for shared environmental health hazards.

**Table 1:** Proposed One Health Competencies for Human Health Professionals

**Skill sets**: • Ability to elicit a history of human-animal-environment interactions. • Inter-professional communication and teamwork skills. • Ability to recognize and treat zoonotic and vector borne disease • Ability to assess and improve patient environments
**Knowledge competencies**: • Zoonotic and vector borne diseases • Animals as sentinels • Human-animal bond and role of service animals, therapy animals, etc. • Prevention of animal-related injuries • Ecosystem function and health • Food systems, in particular animal source foods, in human health and disease • Role of environment on human health • Ethics and values including balance of health and environmental values and legal/ethical limits on physicians dealing with veterinary issues and veterinarians dealing with human health issues • Comparative clinical and evolutionary medicine

Incorporating these competencies into medical education and training will require the design and implementation of innovative curricula in medical education and training.

Such education should include greater instruction on the principles of systems biology and the processes of complex, interdisciplinary problem solving. Academic medical centers can use approaches such as curriculum asset mapping to identify the diverse educational resources necessary to teach such concepts [43]. It will involve expanding the concepts of inter-professional care [44], and education to include animal health and environmental health professionals. Teaching of the patient centered interview would include a greater emphasis on animal contacts and environmental exposures. In the preclinical courses, One Health knowledge competencies could be introduced with lessons drawn from the comparative presentations of particular diseases in different species. These comparative approaches could be reinforced in clinical rotations, with additional reinforcement of history taking skills about animal interactions. Service oriented learning experiences for medical students such as working in clinics serving medically disadvantaged clients could be combined with clinics staffed by veterinary students and attendings for the animals of such clients. Clinical electives could allow medical trainees to shadow veterinarians, while veterinary trainees could spend time shadowing in human health care settings.

### Preliminary steps

The curricula of medical schools are fully packed and it is extremely difficult to introduce new concepts or competencies into the canon. In countries like the US, medical curricula must prepare students to successfully pass standardized board examinations. Since most medical students plan for a career in clinical medicine rather than public health, it is essential to stress the clinical rather than population level applications of the One Health model. Initial attempts to introduce One Health into medical education must recognize and address such constraints. An initial step is to incorporate an introduction to One Health principles in the teaching of zoonotic diseases during infectious disease coursework early in medical school. Educators can demonstrate how One Health approaches can lead to better detection and treatment of such diseases, (some of which will appear on the board examinations). Another preliminary step is to incorporate better animal contact histories into the teaching of clinical interviewing, including the use of standardized patients and recommended history checklists. Clinical electives for interested medical students to explore One Health approaches have been developed at zoos and other facilities near medical schools [45] and show promise for reinforcing One Health concepts during clinical training.

## Conclusion

The One Health approach provides a model for educating medical students and trainees in systems approaches relevant to a range of clinical settings. It also extends traditional concepts of inter-professional education to incorporate animal health and ecosystem aspects of care. Introducing One Health into medical curricula will not be easy but can start with enhanced instruction regarding zoonotic infectious diseases, adding questions about animal contact to the teaching of medical history taking, and creating clinical electives for students to directly experience One Health concepts. Through such innovative approaches, medical students and trainees could acquire clinical One Health competencies enabling them to provide improved patient care and promote healthy environments benefiting all species.
